# Correction: Tree bark scrape fungus: A potential source of laccase for application in bioremediation of non-textile dyes

**DOI:** 10.1371/journal.pone.0245183

**Published:** 2021-01-05

**Authors:** H. M. Bhamare, R. Z. Sayyed, Sapna, Najat Marraiki, Abdallah M. Elgorban, Asad Syed, Hesham Ali El-Enshasy, Daniel J. Dailin

The first two authors, H.M. Bhamare and R.Z. Sayyed, are listed out of order. The first two authors should also be noted as having contributed equally to this work. The third author, Sapna, and the eighth author, Daniel J. Dailin, should be noted as having contributed equally to this work with: Najat Marraiki, Abdallah M. Elgorban, Asad Syed, and Hesham Ali El-Enshasy. Please view the correct author order, affiliations, and citation here:

H. M. Bhamare^1^, R. Z. Sayyed^2^, Sapna^3^, Najat Marraiki^4^, Abdallah M. Elgorban^4,5^, Asad Syed^4^, Hesham Ali El-Enshasy^6,7,8^, Daniel J. Dailin^6,7^

**1** Department of Biotechnology, SSVP Sansth’s Late Karmveer Dr. P. R. Ghogrey Science College, Dhule, India, **2** Department of Microbiology, PSGVP Mandal’s Arts, Science and Commerce College, Shahada, India, **3** ICAR-National Bureau of Plant Genetic Resources, New Delhi, India, **4** Department of Botany and Microbiology, College of Science, King Saud University, Riyadh, Saudi Arabia, **5** Centre of Excellence in Biotechnology Research, King Saud University, Riyadh, Saudi Arabia, **6** Institute of Bioproduct Development, Universiti Teknologi Malaysia (UTM), Skudai, Johor Bahru, Malaysia, **7** School of Chemical and Energy Engineering, Faculty of Engineering, Universiti Teknologi Malaysia (UTM), Skudai, Johor Bahru, Malaysia, **8** City of Scientific Research and Technology Applications, New Burg Al Arab, Alexandria, Egypt

Bhamare HM, Sayyed RZ, Sapna, Marraiki N, Elgorban AM, Syed A, et al. (2020) Tree bark scrape fungus: A potential source of laccase for application in bioremediation of non-textile dyes. PLoS ONE 15(6): e0229968. https://doi.org/10.1371/journal.pone.0229968
